# Association between the use of balanced fluids and outcomes in critically ill children: a before and after study

**DOI:** 10.1186/s13054-021-03705-3

**Published:** 2021-07-29

**Authors:** Matthew F. Barhight, Delphine Nelson, Thomas Moran, Jessica Christiano, L. Nelson Sanchez-Pinto

**Affiliations:** 1 Division of Critical Care, Ann & Robert H. Lurie Children’s Hospital of Chicago, 225 E. Chicago Ave., Chicago, IL 60611 USA; 2 Department of Pediatrics, Northwestern University Feinberg School of Medicine, Chicago, IL USA; 3 Division of Nephrology, Children’s Hospital of Richmond, Richmond, VA USA; 4 Department of Pharmacy, Ann & Robert H. Lurie Children’s Hospital of Chicago, Chicago, IL USA

**Keywords:** Balanced fluids, Acute kidney injury, Mortality, Pediatric critical care

## Abstract

**Background:**

Hyperchloremia and chloride load have been associated with worse clinical outcomes in critically ill patients. We sought to evaluate the electrolyte profile and clinical outcomes associated with a unit-wide transition from saline to balanced fluids for resuscitation and maintenance fluids in a pediatric intensive care unit (PICU).

**Methods:**

A before and after analysis of all patients admitted to the PICU in a large, urban, academic hospital between August 2018 and March 2020. The transition from the use of saline to the use of balanced fluids for both resuscitation and maintenance fluid as standard care occurred in June 2019. The primary outcome was day 3 acute kidney injury (AKI). The secondary outcomes included mortality, ventilator-free days (VFDs), need for renal replacement therapy (RRT), hospital length of stay (LOS), and electrolyte abnormalities.

**Results:**

Overall, 2863 patients (47% female) with a day 3 AKI rate of 12.9% (*n* = 130) and a mortality rate of 2.8% (*n* = 79) were included. After adjusting for confounders (age, PRISM III, mechanical ventilation, and immunocompromised state, septic shock), there were no significant differences in the odds of day 3 AKI (pre 13%, post 12.5%; adjusted odds ratio [aOR] 0.96, 95%CI 0.65–1.42). There were no differences in the secondary outcomes. The post-intervention period had fewer patients with hyperchloremia (pre 15.5% vs. post 10.4%, *p* =  < 0.0001) and hyperkalemia (pre 3.2% vs. post 1.4%, *p* = 0.02) and more patients with hypochloremia (pre 9.5% vs. post 14.4%, *p* =  < 0.0001) and hypokalemia (pre 38.2% vs. post 47.2%, *p* =  < 0.0001). In reference to the normochloremic cohort, the hypochloremic cohort had an increase in day 3 AKI, need for RRT, hyperchloremia, and hyperkalemia, and a decrease in hypokalemia; and the hyperchloremic cohort had an increase in VFD and a decrease in hospital LOS.

**Conclusions:**

Following a unit-wide implementation of balanced fluids as standard care, there were no differences in rates of day 3 AKI or other clinical outcomes. However, there were lower rates of hyperkalemia and hyperchloremia and higher rates of hypokalemia and hypochloremia. Further evaluation of the effect of balanced fluids and the clinical significance of electrolyte abnormalities in critically ill children is needed.

**Supplementary Information:**

The online version contains supplementary material available at 10.1186/s13054-021-03705-3.

## Background

The administration of higher chloride loads and hyperchloremia has been associated with worse clinical outcomes in critically ill patients, including higher rates of mortality and acute kidney injury (AKI) [1–8]. Saline (0.9% sodium chloride fluid) has a considerably larger than the normal concentration of chloride than blood (154 vs. ~ 100 mmol/L) and is one of the most commonly used fluids in the hospital setting [9]. Balanced fluids, like Lactated Ringers or Plasma-Lyte, have more physiologic levels of chloride. While they have been gaining popularity in the last few years, they are still not used as commonly as saline.

In animal studies, elevated tubular chloride leads to afferent arteriolar vasoconstriction, which in turn leads to decreased glomerular filtration rate (GFR) and impaired renal perfusion [10]. Chowdury et al. demonstrated that the administration of hyperchloremic fluid leads to decreased renal blood flow and decreased cortical tissue perfusion in healthy adults [11]. The presence and development of hyperchloremia have been independently associated with higher rates of AKI and mortality in both critically adults and children [4, 5, 8, 12]. Moreover, a recent randomized clinical trial of critically ill adults, Isotonic Solutions and Major Adverse Renal Events Trial, compared the effects of balanced fluids versus saline and found a 1% absolute reduction in the composite outcome of major acute kidney event at 30 days with the use of balanced fluids [6].

AKI occurs commonly in critically ill children, affecting up to 27% of all pediatric intensive care unit (PICU) admissions [13], and is associated with higher mortality, a greater duration of mechanical ventilation, and longer length of stay in both the pediatric and adult the intensive care units [13, 14]. The chloride content of resuscitation and maintenance fluids is a potential modifiable risk factor for AKI, which has led many practitioners to shift toward the use of balanced fluids. In fact, the recent pediatric Surviving Sepsis Campaign guidelines recommend the use of balanced fluids for resuscitation [15]. However, the impact of a change from a saline-based to a balance fluid-based management strategy on the electrolyte profile and outcomes of critically ill children remains unknown.

In this study, we sought to evaluate the electrolyte profiles and clinical outcomes associated with a unit-wide transition from saline to balanced fluids as standard care for resuscitation and maintenance fluid administration in a PICU. We hypothesized that the transition would be associated with improved electrolyte profiles and clinical outcomes.

## Methods

### Study design and population

We performed a before and after study of all patients admitted to the PICU at Ann & Robert H. Lurie Children’s Hospital between August 2018 and March 2020. In June 2019, the pediatric intensive care unit (PICU) transitioned from the use of saline as the main fluid of choice to the use of balanced fluids for both resuscitation and maintenance fluids as standard care. The standard resuscitation fluid was transitioned to a commercially available balanced crystalloid solution (Plasma-Lyte 148), and the standard maintenance fluid became Lactated Ringers with or without dextrose. All care was subject to the preference of the attending physician, and therefore, patients continued to receive saline if there was concern for sodium management or an elevated intracranial pressure. Patients with a primary cardiac diagnosis or a history of congenital heart disease were excluded as they are admitted to a separate cardiac unit. All data were extracted from the electronic health records (EHRs) [16]. The Institutional Review Board at Ann & Robert H. Lurie Children’s Hospital approved the study with a waiver of informed consent.

### Definitions

Patients from August 2018 to March 2019 prior to the clinical change (pre-intervention group) were compared with patients from August 2019 to March 2020 after the clinical change (post-intervention group). The SARS-CoV-2 pandemic occurred after the change to balanced fluids. We therefore limited our data collection to equivalent time-periods prior to March 2020. We collected patient data for the first seven days of hospital stay following admission to the PICU admission. Each PICU day was defined by 24-h periods from the time of admission to the PICU (e.g., day 1 was 0–24 h after admission). *Weight-adjusted fluid volume* was calculated as the total volume administered divided by the PICU admission weight. *Resuscitation fluid* was identified either by the clinical label recorded in the EHR, a single administration of crystalloid or colloid fluid ≥ 10 mL/kg, or was labeled as a “bolus” (e.g., “Normal Saline Bolus”, “Lactated Ringers Bolus”, etc.). Fluid given prior to the admission to the PICU was not included in this assessment. *Severity of illness on admission* was based on their Pediatric Risk of Mortality (PRISM) III score in the first 24 h [17]. *Immunocompromised state* was defined as patients with a malignancy or a history of stem cell or solid organ transplantation [18].

*AKI* was defined using the Kidney Disease Improving Global Outcomes (KDIGO) serum creatinine criteria [19]. Baseline creatinine was defined as the lowest serum creatinine in the 3 months prior to admission. If no baseline creatinine was available, one was calculated using previously validated estimations based on age and sex [20, 21]. *Transient AKI* was defined as AKI on admission to the PICU without AKI on day 3. *Persistent AKI* was defined as AKI on both admission to the PICU and AKI on day 3. *Septic Shock* was defined as known or suspected infection and a receipt of a vasoactive medication.

*Hypochloremia* was defined as a mean chloride level < 98 mmol/L. *Normochloremia* was defined as a mean chloride level 98–109 mmol/L. *Hyperchloremia* was defined as a mean chloride level > 109 mmol/L. *Hyponatremia* was defined as a mean sodium level < 136 mmol/L. *Normonatremia* was defined as a mean sodium level 136–149 mmol/L. *Hypernatremia* was defined as a mean sodium level > 149 mmol/L. *Hypokalemia* was defined as a mean potassium level < 3.9 mmol/L. *Normokalemia* was defined as a mean potassium level 3.9–5.7 mmol/L. *Hyperkalemia* was defined as a mean potassium level > 5.7 mmol/L. *Acidemia* was defined as a mean bicarbonate level < 20 mmol/L. *Alkalemia* was defined as a mean bicarbonate level > 30 mmol/L. Admission levels were based on the first documented laboratory value collected on PICU day 1 and used the same stratifications as described above.

*Total chloride load* was defined as the mmol of chloride per kilogram of weight. *Fluid-adjusted chloride load* was calculated by the chloride load in mmol per kilogram divided by the fluid administered in liters per kilogram. This estimated the type of fluid given based on the fluid-adjusted chloride load (mmol/L), i.e., a patient receiving only saline would have a fluid-adjusted chloride load of 154 mmol/L, those receiving only Plasma-Lyte 148 would have a load of 98 mmol/L, and those receiving only Lactated Ringers would have a load of 109 mmol/L. *Chloride content of maintenance and resuscitation fluid* was calculated through isolation of the fluid-adjusted chloride loads from only maintenance and resuscitation sources. This content was then stratified into hypochloremic (< 98 mmol/L), normochloremic (98–109 mmol/L), and hyperchloremic fluids (> 109 mmol/L).

### Outcomes

The primary outcome was AKI on day 3 of the PICU admission. For this analysis, we included only those patients alive and in the hospital on day 3 following their admission to the PICU. Secondary outcomes included in transient AKI, persistent AKI, hospital mortality, need for renal replacement therapy (RRT), hospital length of stay, ventilator-free days at 28 days, and frequency of electrolyte abnormalities including hyperchloremia, hypochloremia, hypernatremia, hyponatremia, hyperkalemia, hypokalemia, acidemia, and alkalemia. For the secondary outcome analyses, the entire cohort of patients admitted to the PICU were included.

### Sensitivity analyses

We stratified the cohort by the chloride content of maintenance and resuscitation fluids received in the first 3 days of the admission and repeated our outcomes analyses. We also evaluated the effect modification of each of the following on our outcomes of interest: septic shock, cumulative volume of resuscitation fluid (mL/kg) in first 3 days of admission, cumulative total chloride load (mmol/kg) in the first 3 days of admission using both the time-period dichotomy and the chloride content strata. We performed a subpopulation analysis of those patients with a PRISM III score ≥ 10 using both the time-period dichotomy and the chloride content strata. We further performed a subpopulation analysis of those patients who received fluids “per protocol”. We identified the patients who received hyperchloremic maintenance and resuscitation fluids in the pre-intervention time-period and those who received normochloremic fluids in the post-intervention time-period.

Post hoc analyses were performed based on the significant electrolyte abnormalities identified. We evaluated the association of each significantly different electrolyte abnormality (hyperchloremia, hypochloremia, hyperkalemia, hypokalemia) with the outcomes of interest. This was performed because prior literature has demonstrated the association of hyperchloremia with mortality and AKI in critically ill children [7, 8, 12] and the known clinical risks of hyperkalemia and hypokalemia.

We performed at interrupted time-series analysis to see the impact of clinical change over time before and after the clinical change. We evaluated AKI at day 3, mortality, and hospital length of stay.

### Statistical analysis

Data analysis was performed using STATA 14 (StataCorp LP, Texas, USA). Clinical, demographic, and fluid data were summarized and compared between groups. Non-normally distributed continuous variables were compared using Wilcoxon rank-sum and Kruskal–Wallis tests, and categorical variables were compared using the Chi-square test. Following a statistically significant omnibus test, a pairwise comparison using a Bonferroni correction was done as appropriate. Statistical significance level was set at a two-sided alpha < 0.05.

To evaluate the association of the intervention time-period with the outcomes of interest, multivariable logistic regression analyses were performed adjusting for confounders: age, PRISM III score, need for mechanical ventilation, septic shock, and immunocompromised state. The confounding variables were chosen a priori, based on known confounders of mortality. We also repeated the analyses with the addition of admission serum chloride strata using normochloremia as reference. Moreover, the analyses were repeated to evaluate the association of the stratified chloride content of the fluids with the outcomes of interest. We further performed a modified Poisson regression analysis to evaluate the relative risk for developing hyperchloremia and hypochloremia adjusting for the previously stated confounders [22].

The regression analyses previously described were repeated evaluating the effect modification of each of the following on the outcomes of interest: septic shock, volume of resuscitation fluid (mL/kg) during the first 3 days, and chloride load (mmol/kg) during the first 3 days. For the subpopulation analyses (PRISM score ≥ 10 and per protocol) and the electrolyte analyses, we repeated the same outcomes analyses previously described. For the electrolyte analyses, we evaluated hypochloremia, normochloremia, and hyperchloremia (using normochloremia as the reference) and hypokalemia, normokalemia, and hyperkalemia (using normokalemia as the reference), respectively.

We performed an interrupted time-series analysis to evaluate AKI at day 3 and mortality in relation to the intervention time-period. We used an autoregressive integrated moving-average model approach.

## Results

A total of 2863 patients (47% female, *n* = 1345) were included in the study. The patients had a median age of 3.3 years (IQR 1.1–10.4) and a median weight of 14.2 kg (IQR 9.2–29.9). Table 1 presents the clinical characteristics of the study years dichotomized by study time-period (pre- vs. post-intervention). There was a greater proportion of patients requiring mechanical ventilation in the post-intervention time-period. The time-periods had similar admission sources, primary diagnoses, and rates of immunocompromised state and septic shock. When comparing the chloride content of resuscitation and maintenance fluid, patients in the post-intervention time-period received more frequent use of normochloremic fluids and less frequent use of hyperchloremic fluids, but similar use of hypochloremic fluids. Additional file 1: Supplementary Table 1 presents the cumulative administered volumes of each type of fluid in the first 3 days of the PICU stay.

**Table 1:** Demographic, clinical characteristics, and fluid use in the study periods

Demographics	Pre-intervention (*n* = 1483)	Post-intervention (*n* = 1,380)	*p* value
Female *n* (%)	791 (47%)	727 (47%)	0.7
Age (years)	3.2 (1.2–10.4)	3.3 (1–10.4)	0.2
Weight (kg)	14.5 (9.5–30)	14 (8.7–29.9)	0.3
PRISM III	3 (0–7)	3 (0–7)	0.7
*Admission source n (%)*			0.2
Emergency department	546 (36.8%)	473 (34.3%)	
Inpatient	236 (15.9%)	260 (18.8%)	
Operating room	215 (14.5%)	189 (13.7%)	
Outside hospital	486 (32.8%)	458 (33.2%)	
*Primary diagnosis category*			0.06
Respiratory	825 (55.6%)	806 (58.4%)	
Infectious	152 (10.3%)	139 (10.1%)	
Gastrointestinal	40 (2.7%)	54 (3.9%)	
Ingestion	65 (4.4%)	40 (2.9%)	
Cardiovascular	37 (2.5%)	34 (2.5%)	
Endocrine	67 (4.5%)	43 (3.1%)	
Oncologic	85 (5.7%)	74 (5.4%)	
Neurologic	145 (9.8%)	122 (8.8%)	
Orthopedic	35 (2.4%)	21 (1.5%)	
Trauma	16 (1.1%)	27 (2%)	
Renal	16 (1.1%)	20 (1.5%)	
Immunocompromised *n* (%)	170 (11%)	147 (11%)	0.5
Mechanical ventilation *n* (%)	32% (474)	37% (510)	0.005
Septic shock *n* (%)	7% (104)	7% (92)	0.7
*Chloride content of maintenance and resuscitation combined*			< 0.0001
Hypochloremic (< 98 mmol/L)	126 (10%)	139 (11%)	
Normochloremic (98–109 mmol/L)	45 (3.6%)	591 (46.8%)	
Hyperchloremic (> 110 mmol/L)	1087 (86.4%)	532 (41.2%)	
Chloride load from resuscitation days 1–3 (mmol/kg)	3.0 (2.2–4.2) (*n* = 220)	2.0 (1.9–3.1) (*n* = 309)	< 0.0001
Chloride load from maintenance days 1–3 (mmol/kg)	13.5 (4.4–24.5)	10.9 (4.5–18.8)	< 0.0001
*Resuscitation fluid type by patient*	(*n* = 220)	(*n* = 309)	< 0.0001
0.9% Saline	110 (50%)	25 (8%)	
Plasma-Lyte	0 (0%)	271 (88%)	
Lactated Ringers	88 (40%)	4 (1%)	
Combination/Other	22 (10%)	9 (3%)	
*Maintenance Fluid Type per Patient*	(*n* = 1253)	(*n* = 1255)	< 0.0001
0.45% Saline	26 (2%)	19 (1.5%)	
0.675% Saline	3 (0.2%)	35 (3%)	
0.9% Saline	83 (7%)	69 (6%)	
0.9% + 20 mEq KCl	633 (51%)	62 (5%)	
Plasma-Lyte	6 (0.5%)	13 (1%)	
Lactated Ringers	8 (0.6%)	478 (38%)	
Lactated Ringers + 20 mEq KCl	4 (0.3%)	8 (0.6%)	
Combination/Other	490 (39%)	571 (46%)	

The transition from standard fluid practice to the suggested use of Plasma-Lyte as the standard resuscitation fluid and Lactated Ringers as the standard maintenance fluid occurred in June 2019. The pre-intervention time-period era took place from May 2018 to April 2019 and the post-intervention time-period took place from August 2019 to March 2020. Septic shock was defined as the presence of a known infection or concern for an infection and the use of vasoactive medications. Fluid type per patient was estimated by the fluid-adjusted chloride load. This was calculated by the chloride load in mmol per kilogram divided by the fluid administered in liters per kilogram.

The total cohort had a total day 3 AKI rate of 12.9% (*n* = 130), stage 1 AKI 5.8% (*n* = 58), stage 2 AKI 3.6% (*n* = 36), stage 3 AKI 3.6% (*n* = 36), a median hospital length of stay 5.1 days (IQR 2.9–10.5), need for RRT 1.4% (*n* = 39), a mortality rate of 2.8% (*n* = 79), and median ventilator-free days 28 days (IQR 26–28). Table 2 summarizes the outcomes dichotomized by study periods. There was no difference in our primary outcome of day 3 AKI between the two time-periods: pre-intervention 13.3% (*n* = 66) and post-intervention 12.5% (*n* = 64). There was no difference in the rate of persistent AKI or transient AKI between the time-periods. Additional file 1: Supplementary Tables 2 and 3 report the daily AKI stages and rates of severe AKI by time-period for the first 7 days. There were similar rates of RRT and mortality between time-periods. There were longer lengths of stay and fewer ventilator-free days during the post-intervention time-period. In the multivariable analyses, there were no differences in AKI between the pre- and post-intervention time-periods: OR 0.98 (95%CI 0.8–1.3, *p* = 0.9), aOR 1.0 (95%CI 0.8–1.3, *p* = 0.98). Similarly, there were no differences in any of the secondary outcomes in the pre- and post-intervention time-periods.

**Table 2:** Clinical outcomes and electrolyte profiles dichotomized by time-period

Outcomes	Pre-intervention *n* = 1483	Post-intervention *n* = 1380
*Acute kidney injury (day 3) n = 1008*		
*n* (%)	66 (13%)	64 (12.5%)
Unadjusted OR	Ref	0.93 95% CI: 0.64–1.34
Adjusted OR	Ref	0.96 95% CI: 0.65–1.42
*Severe AKI (day 3) n = 1008*		
*n* (%)	34 (6.9%)	38 (7.4%)
Unadjusted OR	Ref	1.08 95% CI: 0.67–1.75
Adjusted OR	Ref	1.17 95% CI: 0.71–1.93
*Mortality*		
*n* (%)	39 (2.6%)	40 (2.9%)
Unadjusted OR	Ref	1.11 95% CI: 0.71–1.73
Adjusted OR	Ref	1.34 95% CI: 0.78–2.31
*Need for renal replacement therapy*		
*n* (%)	15 (1.1%)	24 (1.8%)
Unadjusted OR	Ref	1.65 95% CI: 0.86–3.16
Adjusted OR	Ref	1.9 95% CI: 0.94–3.82
*Hospital length of stay (days)*		
Med (IQR)	4.9 (2.9–9.7)	5.5 (3–11.25)
Unadjusted OR	Ref	1.03 95% CI: 1.01–1.06
Adjusted OR	Ref	1.00 95% CI: 0.98–1.02
*Ventilator-free days*		
Med (IQR)	28 (26–28)	28 (25–28)
Unadjusted OR	Ref	0.99 95% CI: 0.97–1.00
Adjusted OR	Ref	1.00 95% CI: 0.98–1.01
* > 10% Fluid overload on day 3 n = 1008*		
*n* (%)	256 (22%)	355 (30.1%)
Unadjusted OR	Ref	1.54 95% CI: 1.28–1.86
Adjusted OR	Ref	1.56 95% CI: 1.28–1.90
*Hyperchloremia*		
*n* (%)	119 (15.5%)	81 (10.4%)
Unadjusted RR	Ref	0.66 95% CI: 0.51–0.87
Adjusted RR	Ref	0.68 95% CI: 0.53–0.89
*Hypochloremia*		
*n* (%)	73 (9.5%)	112 (14.4%)
Unadjusted RR	Ref	1.51 95% CI: 1.14–1.99
Adjusted RR	Ref	1.49 95% CI: 1.14–1.96
*Hyperkalemia*		
*n* (%)	25 (3.2%)	11 (1.4%)
Unadjusted RR	Ref	0.43 95% CI: 0.21–0.87
Adjusted RR	Ref	0.42 95% CI: 0.21–0.85
*Hypokalemia*		
*n* (%)	296 (38.2%)	373 (47.2%)
Unadjusted RR	Ref	1.23 95% CI: 1.10–1.38
Adjusted RR	Ref	.23 95% CI: 1.10–1.37

Acute kidney injury on day 3 based on KDIGO creatinine definitions. Logistic regression analyses were used to evaluate the intervention periods and day 3 acute kidney injury, mortality and need for renal replacement therapy adjusting for age, PRISM III score, need for mechanical ventilation, and immunocompromised state. Poisson regression analyses were used to evaluate the intervention periods and hospital length of stay and ventilator-free days adjusting for the same confounding variables. Modified Poisson regression analyses were used to evaluate the relative risk for the development of the electrolyte abnormalities.

In the sensitivity analyses, there were no effect modifications due to septic shock, volume of resuscitation (mL/kg), nor total chloride load (mmol/L) in any of the clinical outcomes of interest (day 3 AKI, mortality, or VFD) between the study time-periods. In the subpopulation analyses, patients with a PRISM III score ≥ 10, there was no difference between study periods for day 3 AKI nor mortality. There was a 5% decrease in the likelihood of having a ventilator-free day (aIRR 0.95 95% CI: 0.90–0.99) in the post-intervention period. When we added the admission chloride strata to the model, there continued to be no difference between time-periods. We did find that admission hypochloremia was associated with increased day 3 AKI (2.0 aOR 95%CI: 1.1–3.6) and an increased hospital LOS (1.6 aIRR 95%CI: 1.5–1.6). Admission hyperchloremia was associated with increased day 3 AKI (1.7 aOR 95%CI: 1.1–2.6). In our final subpopulation analysis, “per protocol”, there were no differences between study periods for day 3 AKI, day 3 severe AKI, nor mortality.

Additional file 1: Supplementary Table 4 summarizes the outcomes stratified by the chloride content of maintenance and resuscitation fluids. In the multivariable analyses, the hypochloremic fluid cohort was associated with an increase in day 3 AKI, need for RRT, hyperchloremia, and hyperkalemia and a decrease in hypokalemia. The hyperchloremic fluid cohort was associated with an increase in likelihood of having a ventilator-free day and a decrease in likelihood of an additional hospital day. Both the hypochloremic and hyperchloremic cohorts had an increased risk for the presence of hyperchloremia, but no differences in risk for hypochloremia. Similar to the time-period analysis, there were differences when accounting for the effect modifications of septic shock, volume of resuscitation, or total chloride load.

Additional file 1: Supplementary Table 5 summarizes the electrolyte profiles dichotomized by time-period. The post-intervention period had lower rates of hyperchloremia and hyperkalemia and higher rates of hypochloremia and hypokalemia. In the post hoc analyses of the electrolyte abnormalities, the post-intervention time-period was found to have a lower adjusted risk for both hyperchloremia and hyperkalemia and a higher adjusted risk for both hypochloremia and hypokalemia (Table 2). Table 3 summarizes the daily median serum electrolyte levels for the first 7 days. Additional file 1: Supplementary Figs. 1–4 demonstrate the daily electrolyte levels dichotomized by time-period.

**Table 3:** Daily median electrolytes during first 7 days after admission

Median (IQR)	Pre-intervention	Post-intervention
*Chloride*		
Day 1	108 (104–111)	106 (103–109)*
Day 2	107 (103–110)	105 (101–108)*
Day 3	104 (102–108)	104 (100–107)*
Day 4	104 (100–107)	103 (99–107)*
Day 5	103 (99–107)	103 (99–107)
Day 6	103 (99–107)	102 (98–106)
Day 7	102 (99–106)	102 (98–106)
*Potassium*		
Day 1	4.3 (3.9–4.9)	4.2 (3.8–4.7)*
Day 2	4.1 (3.7–4.6)	3.9 (3.5–4.4)*
Day 3	4.1 (3.7–4.6)	4 (3.5–4.4)*
Day 4	4.1 (3.7–4.5)	3.9 (3.5–4.4)*
Day 5	4.1 (3.8–4.5)	4.1 (3.7–4.5)
Day 6	4.1 (3.7–4.6)	4.1 (3.7–4.6)
Day 7	4.1 (3.8–4.6)	4.2 (3.8–4.6)
*Sodium*		
Day 1	141 (139–144)	141 (139–143)*
Day 2	141 (139–144)	140 (138–143)*
Day 3	141 (138–144)	140 (138–144)
Day 4	140 (138–144)	140 (138–143)
Day 5	140 (137–143)	140 (137–143)
Day 6	140 (137–143)	139 (137–143)
Day 7	140 (137–143)	139 (136–143)
*Bicarbonate*		
Day 1	22 (19.7–25)	21.8 (19.5–24.9)
Day 2	23.1 (20.7–26.5)	23.1 (20.7–26)
Day 3	24.4 (21.8–27.3)	23.9 (21.2–27.1)
Day 4	25 (22.5–29)	24.3 (21.7–28.1)
Day 5	25 (22.2–29)	24 (21.5–29)
Day 6	24.9 (22.4–29.3)	24.5 (21.4–28.5)*
Day 7	25.2 (22.7–29)	24.5 (21.7–29)

*Denotes a statistically significant difference using the Wilcoxon rank-sum test.

Table 4 summarizes the association of the stratified serum chloride levels (hypochloremia, hyperchloremia in reference to normochloremia) with the unadjusted and adjusted outcomes of interest dichotomized by the time-periods. After adjustment for confounders, hypochloremia was associated with higher odds of AKI, and a decreased likelihood of having a ventilator-free day, but not associated with mortality. After adjustment for confounders, hyperchloremia was associated with higher odds of AKI, higher odds of mortality, and a decreased likelihood of having a ventilator-free day. The evaluation of the association of potassium levels with the outcomes of interest indicated that after adjustment for confounders neither hypokalemia nor hyperkalemia was associated with AKI, mortality, or the likelihood of having a ventilator-free day.

**Table 4:** Association between chloride abnormalities and outcomes

Outcomes	Hypochloremia	Normochloremia	Hyperchloremia
*Acute kidney injury (day 3) n = 1008*			
*n* (%)	24 (17.7%)	74 (10.2%)	32 (22.5%)
Unadjusted OR	1.90 95% CI: 1.14–3.13	Ref	2.57 95% CI: 1.62–4.10
Adjusted OR	2.18 95% CI: 1.26–3.77	Ref	2.32 95% CI: 1.39–3.89
*Mortality*			
*n* (%)	11 (6.0%)	30 (2.6%)	25 (12.5%)
Unadjusted OR	2.38 95% CI: 1.17–4.84	Ref	5.40 95% CI: 3.10–9.38
Adjusted OR	1.33 95% CI: 0.59–2.99	Ref	2.84 95% CI: 1.46–5.55
*Need for renal replacement therapy*			
*n* (%)	8 (4.5%)	28 (2.5%)	2 (1.1%)
Unadjusted OR	1.83 95% CI: 0.82–4.07	Ref	0.42 95% CI: 0.10–1.77
Adjusted OR	1.01 95% CI: 0.42–2.46	Ref	0.10 95% CI: 0.02–0.54
*Hospital length of stay (days)*			
Med (IQR)	18.4 (10.6–30.1)	7.7 (4.6–13.9)	8.7 (5–22.5)
Unadjusted IRR	1.69 95% CI: 1.63–1.74	Ref	1.42 95% CI: 1.37–1.47
Adjusted IRR	1.39 95% CI: 1.34–1.43	Ref	1.26 95% CI: 1.21–1.30
*Ventilator-free days*			
Med (IQR)	21 (13–28)	28 (24–28)	27 (17–28)
Unadjusted IRR	0.77 95% CI: 0.74–0.80	Ref	0.84 95% CI: 0.81–0.86
Adjusted IRR	0.89 95% CI: 0.86–0.92	Ref	0.93 95% CI: 0.90–0.96

*Hypochloremia* was defined as < 98 mmol/L. *Normochloremia* was defined as 98–109 mmol/L. *Hyperchloremia* was defined as > 109 mmol/L. Logistic regression analyses were used to evaluate hyperchloremia and hypochloremia in reference to normochloremia with acute kidney injury, mortality, and need for renal replacement therapy adjusting for age, PRISM III score, need for mechanical ventilation, and immunocompromised state. Poisson regression analysis was used to evaluate hyperchloremia and hypochloremia in reference to normochloremia with hospital length of stay and ventilator-free days adjusting for the same confounding variables.

We performed an interrupted time-series analysis for both day 3 AKI and mortality. There was no difference between time-periods for either outcome of interest. Additional file 1: Supplementary Fig. 5 demonstrates the day 3 AKI rate over time, and Additional file 1: Supplementary Fig. 6 demonstrates the mortality rate over time.

## Discussion

In this before and after study, we evaluated the clinical outcomes following a change from saline to balanced fluids as standard care for resuscitation and maintenance fluids for patients admitted to a large urban tertiary care PICU. To our knowledge, this study represents the first evaluation of balanced fluids as standard care in a PICU. The chloride content of maintenance and resuscitation fluids administered demonstrated the transition from the frequent use of hyperchloremic fluids to the use of normochloremic fluids in the post-intervention period. Overall, this increased use of balanced fluids was associated with a lower chloride load administered to patients. Most of this decreased load was attributable to the change in the use of balanced maintenance fluids. Despite the changes in fluid type and chloride loads, there were no significant differences in the primary and secondary outcomes between the study time-periods, including AKI, in-hospital mortality, need for RRT, hospital length of stay, or ventilator-free days. There was, however, a significant change in the profile of electrolyte abnormalities, with lower rates of hyperchloremia and hyperkalemia and higher rates of hypochloremia and hypokalemia in the post-intervention period. Furthermore, the presence of chloride abnormalities was independently associated with worse clinical outcomes.

We also evaluated the clinical outcomes stratified by the chloride content of the fluids used regardless of time-period. The use of hypochloremic fluids was associated with an increase in day 3 AKI and need for RRT; the use of hyperchloremic fluids was associated with an increase in the likelihood of having a ventilator-free day and a decrease in the likelihood of an additional hospital day. However, it is likely that these findings reflect cofounding by indications rather than a causal relationship, and these results should be viewed cautiously and as hypothesis-generating. Most patients with hypochloremic fluids in our ICU receive acetate instead of chloride in their maintenance fluids. This practice is reserved for children with a higher severity of illness and metabolic acidosis.

Several randomized control trials of balanced fluids vs. saline in adult patients have also shown no difference in outcomes. The saline vs. Plasma-Lyte for ICU fluid therapy trial was a randomized control trial that evaluated critically ill adults and compared the use of saline vs. Plasma-Lyte 148. The authors demonstrated no significant difference in outcomes between the two study groups [23]. The saline versus Lactated Ringers randomized control trial evaluated adults receiving non-emergent abdominal surgery and compared the two crystalloid fluid types during the operation [24]. The investigators found no differences in clinical outcomes. In contrast to these findings, the Isotonic Solutions and Major Adverse Renal Events Trial showed improvements in the composite outcome of mortality, new need for RRT, and persistent reduction in renal function among patients receiving balanced fluids [6]. There are important differences between each of these studies notably the severity of illness of each population, and the administration of saline to patients prior to initiation of the studies. These two issues likely affect our findings as well. We included a heterogeneous population of children typical of a general PICU, including many children with a low severity of illness (i.e., median PRISM III score of 3). However, in our sensitivity analyses of only those patients with PRISM III scores ≥ 10, we found results similar to our full cohort analyses.

Our findings as they relate to the patient electrolyte abnormality profiles are notable. In the post-intervention time-period, there were lower rates of hyperchloremia and hyperkalemia as well as higher rates of hypochloremia and hypokalemia. In the pre-intervention time-period, there was common use of saline with 20 mEq/L of potassium chloride for maintenance fluid. The decrease in both chloride and potassium likely was the driver of these changes. Importantly, there were no differences in the sodium or bicarbonate abnormalities. In the post hoc analyses of these electrolyte abnormalities, hyperchloremia was associated with a higher rate of AKI, a higher rate mortality and a lower likelihood of having a ventilator-free day after adjusting for confounders, and hypochloremia was associated with a higher rate of AKI and lower likelihood of having a ventilator-free day. This association of serum chloride levels and poor clinical outcomes is consistent with prior literature in critically ill adults and children [2, 7, 8, 12, 25, 26]. However, our findings are notable for the significant change in the electrolyte abnormality profiles between the two time-periods, and the transition to balanced fluids did not lead to more normalized electrolyte homeostasis. The clinical relevance of these electrolyte changes is undetermined. Further study is needed to elucidate the impact chloride content and chloride load have on patients with hyperchloremia and hypochloremia and their associated outcomes. A potential future study could use an electrolyte-driven fluid protocol.

This study has several strengths. The study cohort is large and heterogeneous. In addition, the comparison groups are from similar time-periods and represent similar populations. The study also has several limitations. First, the data are retrospective and observational, and the study design can only establish associations and not causality. Second, we assessed the chloride load from all intravenous sources, but were unable to assess the chloride load from enteral sources. Third, these results could be subject to a selection bias due to the types of patients admitted to a large urban pediatric hospital. Fourth, the SARS-CoV-2 pandemic impacted our hospital starting in March 2020 and limited the ability to compare a larger number of patients and likely limited the power to detect possible differences between the study periods. Fifth, though the only major change that occurred in the PICU during the study period was the increased use of balanced fluids, other unmeasured changes may have influenced the outcomes. Sixth, due to the concurrent timing of the AKI and electrolyte abnormalities, it is impossible to assess the directionality of effect. Last, patients may have received fluids of potentially large volumes prior to coming to the PICU. The composition of fluids prior to admission was not calculated and may have influenced the outcomes. Anecdotally, the most used fluid in other parts of the hospital was and remains saline.

## Conclusions

Following a practice change in a PICU from the use of saline to the use of balanced fluids as standard care for both maintenance fluids and resuscitation, there were no significant differences in clinical outcomes including day 3 AKI and mortality. However, the intervention was associated with a significant change in the profile of electrolyte abnormalities. The balanced fluid time-period was associated with significantly lower rates of hyperkalemia and hyperchloremia, but with higher rates of hypokalemia and hypochloremia. Both hyper- and hypochloremia were independently associated with increased rates of day 3 AKI, decreased ventilator-free days after adjustment for confounders. Hyperchloremia was also independently associated with higher in-hospital mortality. The direct impact of the use of balanced fluids on the profiles of electrolyte abnormalities and its association with outcomes in critically ill children requires further evaluation.

## Supplementary Information

**Additional file 1**. Supplementary Tables/Figures.

## Data Availability

The datasets used and/or analyzed during the current study are available from the corresponding author on reasonable request.

## Abbreviations

AKI: Acute kidney injury
PICU: Pediatric intensive care unit
EHR: Electronic health record
GFR: Glomerular filtration rate
OR: Odds ratio
VFD: Ventilator-free days
RRT: Renal replacement therapy
PRISM: Pediatric risk of mortality

## References

[1] Lee JY, Hong TH, Lee KW, Jung MJ, Lee JG, Lee SH (2016). Hyperchloremia is associated with 30-day mortality in major trauma patients: a retrospective observational study. Scand J Trauma Resusc Emerg Med.

[2] Zhang Z, Xu X, Fan H, Li D, Deng H (2013). Higher serum chloride concentrations are associated with acute kidney injury in unselected critically ill patients. BMC Nephrol.

[3] Toyonaga Y, Kikura M (2017). Hyperchloremic acidosis is associated with acute kidney injury after abdominal surgery. Nephrology (Carlton).

[4] Suetrong B, Pisitsak C, Boyd JH, Russell JA, Walley KR (2016). Hyperchloremia and moderate increase in serum chloride are associated with acute kidney injury in severe sepsis and septic shock patients. Crit Care.

[5] Neyra JA, Canepa-Escaro F, Li X, Manllo J, Adams-Huet B, Yee J, Yessayan L (2015). Group AKIiCIS: association of hyperchloremia with hospital mortality in critically ill septic patients. Crit Care Med.

[6] Semler MW, Self WH, Wanderer JP, Ehrenfeld JM, Wang L, Byrne DW, Stollings JL, Kumar AB, Hughes CG, Hernandez A (2018). Balanced crystalloids versus saline in critically ill adults. N Engl J Med.

[7] Barhight MF, Lusk J, Brinton J, Stidham T, Soranno DE, Faubel S, Goebel J, Mourani PM, Gist KM (2018). Hyperchloremia is independently associated with mortality in critically ill children who ultimately require continuous renal replacement therapy. Pediatr Nephrol.

[8] Barhight MF, Brinton J, Stidham T, Soranno DE, Faubel S, Griffin BR, Goebel J, Mourani PM, Gist KM (2018). Increase in chloride from baseline is independently associated with mortality in critically ill children. Intensive Care Med.

[9] Kellum JA (2002). Fluid resuscitation and hyperchloremic acidosis in experimental sepsis: improved short-term survival and acid-base balance with Hextend compared with saline. Crit Care Med.

[10] Wilcox CS (1983). Regulation of renal blood flow by plasma chloride. J Clin Investig.

[11] Chowdhury AH, Cox EF, Francis ST, Lobo DN (2012). A randomized, controlled, double-blind crossover study on the effects of 2-L infusions of 0.9% saline and plasma-lyte® 148 on renal blood flow velocity and renal cortical tissue perfusion in healthy volunteers. Ann Surg.

[12] Stenson EK, Cvijanovich NZ, Anas N, Allen GL, Thomas NJ, Bigham MT, Weiss SL, Fitzgerald JC, Checchia PA, Meyer K (2018). Hyperchloremia is associated with complicated course and mortality in pediatric patients with septic shock. Pediatr Crit Care Med.

[13] Kaddourah A, Basu RK, Bagshaw SM, Goldstein SL, Investigators A (2016). Epidemiology of acute kidney injury in critically ill children and young adults. N Engl J Med.

[14] Hoste EA, Bagshaw SM, Bellomo R, Cely CM, Colman R, Cruz DN, Edipidis K, Forni LG, Gomersall CD, Govil D (2015). Epidemiology of acute kidney injury in critically ill patients: the multinational AKI-EPI study. Intensive Care Med.

[15] Weiss SL, Peters MJ, Alhazzani W, Agus MSD, Flori HR, Inwald DP, Nadel S, Schlapbach LJ, Tasker RC, Argent AC (2020). Executive summary: surviving sepsis campaign international guidelines for the management of septic shock and sepsis-associated organ dysfunction in children. Intensive Care Med.

[16] Kahn MG, Callahan TJ, Barnard J, Bauck AE, Brown J, Davidson BN, Estiri H, Goerg C, Holve E, Johnson SG (2016). A harmonized data quality assessment terminology and framework for the secondary use of electronic health record data. EGEMS (Wash DC).

[17] Pollack MM, Patel KM, Ruttimann UE (1996). PRISM III: an updated Pediatric Risk of Mortality score. Crit Care Med.

[18] Feudtner C, Feinstein JA, Zhong W, Hall M, Dai D (2014). Pediatric complex chronic conditions classification system version 2: updated for ICD-10 and complex medical technology dependence and transplantation. BMC Pediatr.

[19] Khwaja A (2012). KDIGO clinical practice guidelines for acute kidney injury. Nephron Clin Pract.

[20] Hessey E, Ali R, Dorais M, Morissette G, Pizzi M, Rink N, Jouvet P, Lacroix J, Phan V, Zappitelli M (2017). Evaluation of height-dependent and height-independent methods of estimating baseline serum creatinine in critically ill children. Pediatr Nephrol.

[21] Hoste L, Dubourg L, Selistre L, De Souza VC, Ranchin B, Hadj-Aissa A, Cochat P, Martens F, Pottel H (2014). A new equation to estimate the glomerular filtration rate in children, adolescents and young adults. Nephrol Dial Transplant.

[22] Zou G (2004). A modified poisson regression approach to prospective studies with binary data. Am J Epidemiol.

[23] Young P, Bailey M, Beasley R, Henderson S, Mackle D, McArthur C, McGuinness S, Mehrtens J, Myburgh J, Psirides A (2015). Effect of a Buffered crystalloid solution vs saline on acute kidney injury among patients in the intensive care unit: The SPLIT randomized clinical trial. JAMA.

[24] Maheshwari K, Turan A, Makarova N, Ma C, Esa WAS, Ruetzler K, Barsoum S, Kuhel AG, Ritchey MR, Higuera-Rueda C (2020). Saline versus lactated Ringer's solution: the saline or lactated Ringer's (SOLAR) trial. Anesthesiology.

[25] de Vasconcellos K, Skinner DL (2018). Hyperchloraemia is associated with acute kidney injury and mortality in the critically ill: a retrospective observational study in a multidisciplinary intensive care unit. J Crit Care.

[26] Neyra JA, Canepa-Escaro F, Li X, Manllo J, Adams-Huet B, Yee J, Yessayan L (2015). Acute kidney injury in critical illness study G: Association of Hyperchloremia with hospital mortality in critically ill septic patients. Crit Care Med.

