# Supplementary material for: Association between the use of balanced fluids and outcomes in critically ill children: a before and after study
Source: Crit Care. 2021 Jul 29;25:266. doi: 10.1186/s13054-021-03705-3 (PMC8319885; doi:10.1186/s13054-021-03705-3)
Supplement: Supplementary file 1 — Additional file 1. Supplementary Tables/Figures. [file 13054_2021_3705_MOESM1_ESM.pdf]

Supplementary Tables and Figures

Table of Contents:

Supplementary Table 1: Cumulative Fluid Administered Dichotomized by time-periods: page 2

Supplementary Table 2: Daily Stages of AKI by Time Period: page 3

Supplementary Table 3: Daily Severe AKI by Time Period: page 4

Supplementary Table 4: Clinical Outcomes Stratified by Chloride Content of Resuscitation and Maintenance: pages 5-6

Supplementary Table 5: Electrolyte profiles and abnormalities in the first 7 days after admission: page 7-8

Supplementary Figure 1: Daily Chloride for first 7 days: page 9

Supplementary Figure 2: Daily Sodium for first 7 days: page 10

Supplementary Figure 3: Daily Potassium for first 7 days: page 11

Supplementary Figure 4: Daily Bicarbonate for first 7 days: page 12

Supplementary Figure 5: Interrupted Time Series Day 3 AKI: page 13

Supplementary Figure 6: Interrupted Time Series Mortality: page 14

Supplementary Table 1: Cumulative Fluid Administered in First 3 Days Dichotomized by time-periods

Fluid Type (mL/kg)		Pre-Intervention	Post-Intervention	p value
Resuscitation	mean ± SD	5 ± 18	6 ± 14	<0.0001
	median (IQR)	0 (0-0)	0 (0-0)	
Maintenance	mean ± SD	114 ± 89	112 ± 84	0.84
	median (IQR)	99.3 (44.8-171.7)	98.2 (47.3-163.7)	
Blood Products	mean ± SD	2 ± 42	1 ± 7	0.4
	median (IQR)	0 (0-0)	0 (0-0)	
Fluid Creep	mean ± SD	7 ± 13	11 ± 20	<0.0001
	median (IQR)	0.6 (0-7.4)	1.4 (0-14)	
Nutrition	mean ± SD	98 ± 92	103 ± 95	0.25
	median (IQR)	69.9 (25.7-148.8)	73.6 (24.6-159.8)	
RRT Volume	mean ± SD	3 ± 33	4 ± 49	0.34
	median (IQR)	0 (0-0)	0 (0-0)	

Legend: The cumulative totals for each fluid type during the first 3 days following PICU admission. All volumes reported are in mL/kg.

Supplementary Table 2: Daily Stages of AKI by Time Period

Stage of AKI	Day 1 n= 1831		Day 2 n=1244		Day 3 n=1008		Day 4 n=839		Day 5 n=737		Day 6 n=653		Day 7 n=552	
	Pre	Post	Pre	Post	Pre	Post	Pre	Post	Pre	Post	Pre	Post	Pre	Post
0	824 (87.7%)	772 (86.6%)	542 (87.2%)	553 (88.6%)	429 (86.7%)	449 (87.5%)	351 (85.4%)	380 (99.8%)	316 (85.6%)	323 (87.5%)	275 (86.2%)	292 (87.4%)	239 (86.3%)	236 (85.8%)
1	60 (6.4%)	56 (6.3%)	40 (6.5%)	34 (5.5%)	32 (6.5%)	26 (5.1%)	28 (6.8%)	18 (4.2%)	28 (7.6%)	20 (5.4%)	24 (7.5%)	14 (4.2%)	19 (6.9%)	16 (5.8%)
2	26 (2.8%)	28 (3.1%)	18 (2.9%)	12 (1.9%)	18 (3.6%)	18 (3.5%)	17 (4.1%)	9 (2.1%)	9 (2.5%)	8 (2.2%)	7 (2.2%)	12 (3.6%)	7 (2.5%)	9 (3.3%)
3	30 (3.2%)	35 (3.9%)	20 (3.2%)	25 (4.0%)	16 (3.2%)	20 (3.9%)	15 (3.7%)	21 (4.9%)	15 (4.1%)	18 (4.9%)	13 (4.1%)	16 (4.8%)	12 (4.3%)	14 (5.1%)

Supplementary Table 3: Daily Severe AKI by Time Period

	Day 1<br>n= 1831	Day 2<br>n=1244	Day 3<br>n=1008	Day 4<br>n=839	Day 5<br>n=737	Day 6<br>n=653	Day 7<br>n=552
Pre-Intervention	56 (6%)<br>Ref	38 (6.1%)<br>Ref	34 (6.9%)<br>Ref	32 (7.8%)<br>Ref	24 (6.5%)<br>Ref	20 (6.3%)<br>Ref	19 (6.9%)<br>Ref
Post-Intervention	63 (7.1%)<br>OR: 1.3<br>95% CI: 0.88-1.97	37 (5.9%)<br>OR: 1.03<br>95% CI: 0.63-1.69	38 (7.4%)<br>OR: 1.17<br>95% CI: 0.71-1.94	30 (7%)<br>OR: 0.92<br>95% CI: 0.53-1.59	26 (7.1%)<br>OR: 1.12<br>95% CI: 0.62-2.02	28 (8.4%)<br>OR: 1.45<br>95% CI: 0.79-2.67	23 (8.4%)<br>OR 1.31<br>95% CI: 0.69-2.52

Supplementary Table 4: Outcomes by Chloride Content of IV Resuscitation and Maintenance Fluids

Outcomes	Hypochloremic Fluid	Normochloremic Fluid	Hyperchloremic Fluid
Acute Kidney Injury (Day 3) n=1008			
n(%)	59 (34.3%)	18 (9%)	44 (7.8%)
Unadjusted OR	5.3 95% CI: 3.0-9.4	Ref	0.9 95% CI: 0.5-1.5
Adjusted OR	3.9 95% CI: 1.2-7.1	Ref	0.8 95% CI: 0.4-1.3
Mortality			
n(%)	24 (9.1%)	17 (2.7%)	32 (2%)
Unadjusted OR	3.6 95% CI: 1.9-6.9	Ref	0.7 95% CI: 0.4-1.3
Adjusted OR	1.0 95% CI: 0.4-2.4	Ref	0.6 95% CI:0.3-1.3
Need for Renal Replacement Therapy			
n(%)	17 (6.4%)	6 (0.9%)	15 (0.9%)
Unadjusted OR	7.2 95% CI: 2.8-18.5	Ref	1.0 95% CI: 0.4-2.5
Adjusted OR	4.0 95% CI: 1.4-11.0	Ref	1.1 95% CI: 0.4-2.8
Hospital Length of Stay (days)			
Med (IQR)	8.1 (3.8-20.1)	4.9 (2.9-10.0)	5.1 (3-10)
Unadjusted IRR	1.7 95% CI: 1.6-1.8	Ref	0.94 95% CI: 0.91-0.96
Adjusted IRR	1.33 95% CI: 1.28-1.38	Ref	0.94 95% CI: 0.92-0.97
Ventilator Free Days			
Med (IQR)	28 (23-28)	28 (26-28)	28 (26-28)
Unadjusted IRR	0.95 95% CI: 0.92-0.98	Ref	1.02 95% CI: 1.01-1.04
Adjusted IRR	1.02 95% CI: 0.99-1.05	Ref	1.02 95% CI: 1.0-1.03
% Fluid Overload on Day 3 n=1008			
n(%)	82 (33.3%)	164 (29.7%)	326 (23.3%)
Unadjusted OR	1.19 95% CI: 0.86-1.64	Ref	0.72 95% CI: 0.58-0.90
Adjusted OR	1.30 95% CI: 0.91-1.86	Ref	0.73 95% CI: 0.58-0.92
Hyperchloremia			
n(%)	50 (23.2%)	23 (7.1%)	111 (12.7%)
Unadjusted RR	3.26 95% CI: 2.05-5.18	Ref	2.79 95% CI: 1.74-4.50
Adjusted RR	1.78 95% CI: 1.16-2.74	Ref	1.84 95% CI: 1.20-2.82
Hypochloremia			

n(%)	35 (16.2%)	47 (14.5%)	82 (9.4%)
Unadjusted RR	1.11 95% CI: 0.75-1.67	Ref	0.65 95% CI: 0.46-0.90
Adjusted RR	1.09 95% CI: 0.71-1.66	Ref	0.67 95% CI: 0.48-0.92
Hyperkalemia			
n(%)	7 (3.2%)	1 (0.3%)	26 (3.0%)
Unadjusted RR	10.5 95% CI: 1.30-85.1	Ref	9.67 95% CI: 1.31-71.0
Adjusted RR	16.0 95% CI: 2.07-124.3	Ref	9.94 95% CI: 1.35-73.4
Hypokalemia			
n(%)	105 (48.2%)	169 (51.5%)	346 (39.2%)
Unadjusted RR	0.93 95% CI: 0.79-1.11	Ref	0.76 95% CI: 0.67-0.87
Adjusted RR	0.81 95% CI: 0.68-0.97	Ref	0.75 95% CI: 0.66-0.85

Legend: Chloride content of maintenance and resuscitation fluid was calculated through isolation of the fluid adjusted chloride loads from only maintenance and resuscitation sources. This content was then stratified into hypochloremic (<98 mmol/L), normochloremic (98-109 mmol/L), and hyperchloremic fluids (>109 mmol/L). AKI on day 3 is based on KDIGO creatinine definitions. Logistic regression analyses were used to evaluate the chloride content stratification using normochloremic fluids as reference and day 3 acute kidney injury, mortality and need for renal replacement therapy adjusting for age, PRISM III score, need for mechanical ventilation, and immunocompromised state. Poisson regression analyses were used to evaluate the chloride content stratification and length of stay and ventilator free days adjusting for the same confounding variables. Modified Poisson regression analyses were used to evaluate the relative risk for the development of the electrolyte abnormalities.

Supplementary Table 5: Electrolyte profiles and abnormalities in the first 7 days after admission.

Electrolytes	Pre-intervention	Post-intervention	p value
**Weekly Mean**			
**Chloride**			
Hyperchloremia	119 (15.5%)	81 (10.4%)	<0.0001
Normochloremia	575 (75%)	587 (75%)	
Hypochloremia	73 (9.5%)	112 (14.4%)	
**Potassium**			
Hyperkalemia	25 (3.2%)	11 (1.4%)	<0.0001
Normokalemia	453 (58.5%)	407 (51.5%)	
Hypokalemia	296 (38.2%)	373 (47.2%)	
**Sodium**			
Hypernatremia	37 (4.7%)	36 (4.5%)	0.27
Normonatremia	697 (88.6%)	689 (86.6%)	
Hyponatremia	53 (6.7%)	71 (8.9%)	
**Bicarbonate**			
Low/Acidosis	144 (18%)	144 (18.2%)	0.96
Normal	553 (71.3%)	565 (71.5%)	
High/Alkalosis	83 (10.7%)	164 (10.5%)	
**On Admission**			
**Chloride**			
Hyperchloremia	200 (21.3%)	80 (9%)	<0.0001
Normochloremia	687 (73.3%)	703 (76.9%)	
Hypochloremia	50 (5.3%)	80 (9%)	
**Potassium**			
Hyperkalemia	41 (4.4%)	38 (4.2%)	<0.0001
Normokalemia	500 (53%)	406 (44.4%)	
Hypokalemia	402 (42.6%)	470 (51.4%)	
**Sodium**			
Hypernatremia	47 (4.9%)	43 (4.7%)	<0.0001

Normonatremia	796 (83.4%)	703 (76.9%)	
Hyponatremia	111 (11.6%)	168 (18.4%)	
**Bicarbonate**			
Low/Acidosis	384 (40.6%)	413 (45%)	0.1
Normal	534 (56.5%)	473 (51.6%)	
High/Alkalosis	28 (3%)	31 (3.4%)	

Legend: *Hypochloremia* was defined as <98mmol/L. *Normochloremia* was defined as 98-109mmol/L. *Hyperchloremia* was defined as >109mmol/L. *Hyponatremia* was defined as <136mmol/L. *Normonatremia* was defined as 136-149mmol/L. *Hypernatremia* was defined as >149mmol/L. *Hypokalemia* was defined as <3.9mmol/L. *Normokalemia* was defined as 3.9-5.7mmol/L. *Hyperkalemia* was defined as >5.7mmol/L. *Acidemia* was defined as <20mmol/L of bicarbonate. *Alkalemia* was defined as >30 mmol/L of bicarbonate.

Supplementary Figure 1: Daily Chloride for First 7 Days Dichotomized by Time Period

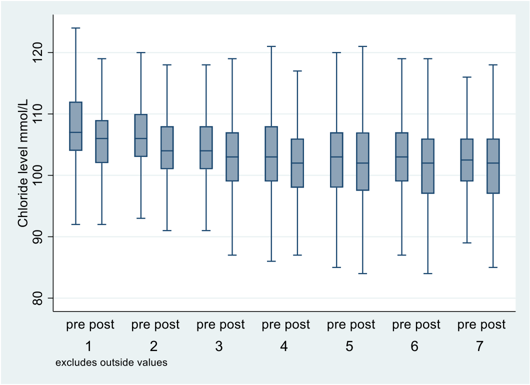

Legend: Reporting the median daily chloride levels for each patient.

Supplementary Figure 2: Daily Sodium for First 7 Days Dichotomized by Time Period

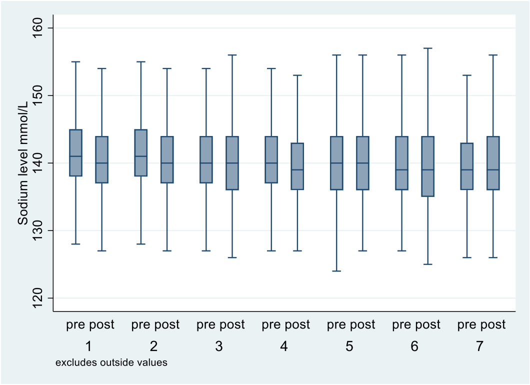

Legend: Reporting the median daily sodium levels for each patient.

Supplementary Figure 3: Daily Potassium for First 7 Days Dichotomized by Time Period

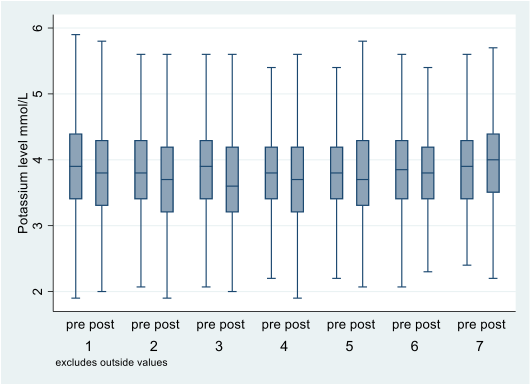

Legend: Reporting the median daily potassium levels for each patient.

Supplementary Figure 4: Daily Bicarbonate for First 7 Days Dichotomized by Time Period

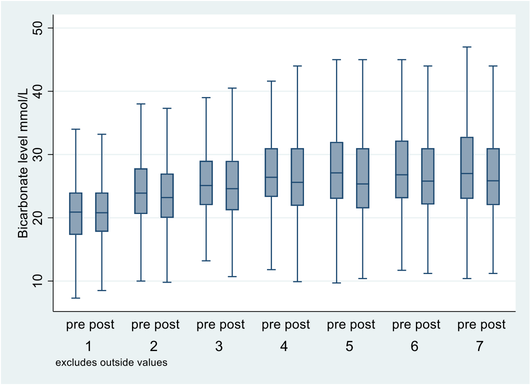

Legend: Reporting the median daily bicarbonate levels for each patient.

Supplementary Figure 5: Interrupted Time Series Day 3 AKI

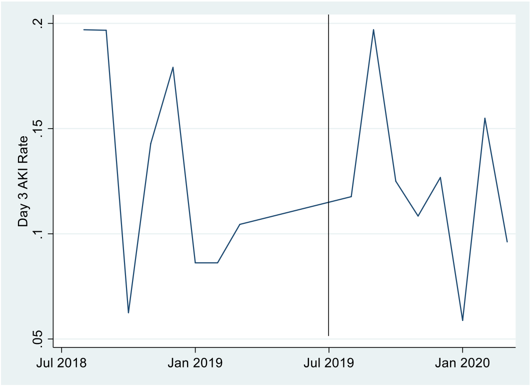

Legend: The monthly day 3 AKI rate is reported. The vertical line denotes the intervention.

Supplementary Figure 6: Interrupted Time Series Mortality

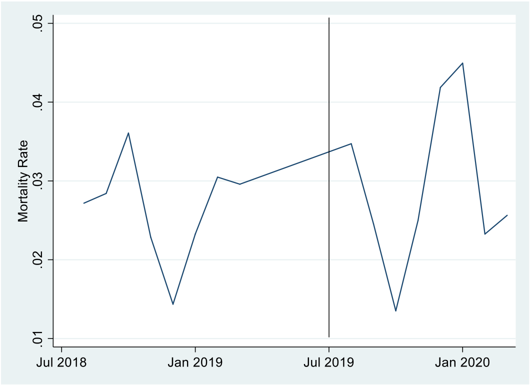

Legend: The monthly mortality rate is reported. The vertical line denotes the intervention.
